# Salivary creatinine and urea are higher in an experimental model of acute but not chronic renal disease

**DOI:** 10.1371/journal.pone.0200391

**Published:** 2018-07-06

**Authors:** Alexandra Kovalčíková, Katarína Janšáková, Marianna Gyurászová, Ľudmila Podracká, Katarína Šebeková, Peter Celec, Ľubomíra Tóthová

**Affiliations:** 1 Institute of Molecular Biomedicine, Faculty of Medicine, Comenius University, Bratislava, Slovakia; 2 1st Department of Pediatrics, Faculty of Medicine, Comenius University, Bratislava, Slovakia; 3 Institute of Pathophysiology, Faculty of Medicine, Comenius University, Bratislava, Slovakia; 4 Department of Molecular Biology, Faculty of Natural Sciences, Comenius University, Bratislava, Slovakia; University of Sao Paulo Medical School, BRAZIL

## Abstract

Plasma creatinine and urea are commonly used markers of kidney function in both acute and chronic renal failure. The needed repeated blood collection is associated with pain, stress and might lead to infections. Saliva has the potential to be a non-invasive alternative diagnostic fluid. The use of saliva in clinical practice is limited, since many factors affect the concentration of salivary biomarkers. The aim of our study was to analyze salivary creatinine and urea in the animal models of acute and chronic renal disease. Bilateral nephrectomy and adenine nephropathy were induced in adult male mice. Both, plasma creatinine and urea were higher in animals with renal failure compared to controls. Salivary creatinine was higher by 81% and salivary urea by 43% in comparison to the control group, but only in animals with bilateral nephrectomy and not in adenine nephropathy. Our results indicate that the increase of salivary creatinine and urea depends on the experimental model of renal failure and its severity. Further studies are needed to monitor the dynamics of salivary markers of renal function and to reveal determinants of their variability.

## Introduction

The prevalence of acute kidney injury (AKI) and chronic kidney disease (CKD) is increasing at alarming rates [1,2]. Assessment of plasma creatinine and urea concentration using standard biochemical methods is a common approach for the evaluation of kidney function in clinical practice [3]. Creatinine, a metabolite of phosphocreatine diffuses from various tissues into blood. Under physiological circumstances, it is constantly excreted via kidneys. Impaired glomerular filtration leads to a rise in plasma creatinine. Urea is produced by the liver and excreted by the kidneys. Similarly to creatinine, an increase of plasma urea serves as an indicator of altered kidney functions. However, concentrations of creatinine and urea could be affected by food intake, drugs or intestinal bacteria [4–6].

Blood collection is widely used and standardized, but especially in children and aged people it is associated with stress and pain. Repeated blood collections can lead to a higher risk of infections. Collection of alternative body fluids such as saliva is in comparison to blood easy, non-invasive and can be conducted even at home [7]. Accumulation of creatinine in the blood due to kidney disease leads to increasing concentration gradient that facilitates diffusion to saliva. Moreover, high plasma creatinine increases the permeability of salivary glands [8,9]. Urea has a low molecular weight and can easily undergo ultrafiltration from blood to saliva [9]. Thus, saliva collection might represent a non-invasive alternative to blood collection for diagnostics and monitoring of kidney disease. Collection of saliva is cheaper and does not require trained personnel. The use of saliva in clinical practice would eliminate or at least reduce problems associated with repeated blood collection. In addition, patients could collect their saliva samples at home without stress induced by health care providers [10].

Several studies have found higher salivary creatinine in patients with CKD compared to age-matched healthy controls. Additionally, a positive correlation between serum/plasma and salivary creatinine concentrations was confirmed, at least in CKD patients [11–13]. Similar results were obtained for salivary urea [14]. In addition, patients with severe kidney damage had higher salivary creatinine and urea compared to patients with moderate kidney damage. A positive correlation was found between severity of kidney disease and concentration of creatinine and urea [13,15].

Creatinine and urea in saliva are influenced by many determinants that lead to high technical and biological variability. Bias that prevent the use of salivary markers in routine medical practice might arise from eating, drinking, tooth brushing or blood contamination but also from yet unidentified factors [16,17]. In addition, bacterial ureases hydrolyse urea to ammonia and carbon dioxide [18]. Changes in the urea excretion rate of individual glands could influence the concentration of salivary urea. Saliva from the parotid gland has significantly higher concentration of urea compared to saliva from submandibular and sublingual glands [19]. In addition, the effects of some drugs on salivary urea concentration were described [20,21].

Since it is difficult to eliminate bias in human studies, the animal models are an important tool to study factors influencing the variability of salivary biomarkers.

One of the commonly used surgical models with a rapid increase of serum creatinine is bilateral nephrectomy (BNX) [22]. Adenine nephropathy is a chemically induced animal model of CKD, in which crystals are formed in tubules [23]. Supplementation of a large amount of adenine leads to its oxidation to 2,8-dihydroxyadenine via xanthine dehydrogenase. At the physiological pH of urine, 2,8-dihydroxyadenine is only poorly soluble, which leads to its precipitation and deposition [24].

It is still not clear whether salivary creatinine and urea reflect their plasma levels at any stage of renal disease or whether they need to reach a certain threshold in plasma to diffuse to saliva. To the best of our knowledge, a study evaluating salivary creatinine and urea in mice with induced BNX or adenine nephropathy has not been published yet. Therefore, the aim of this experiment was to evaluate whether the loss of renal function in experimental models of AKI and CKD would be reflected also by elevation of salivary creatinine and urea. BNX and adenine nephropathy were used, as they mimic different types and severities of renal failure.

## Methods

### Animals

This study was approved by the ethics committee of the Institute of Molecular Biomedicine, Comenius University and was carried out according to relevant national legislation. In this study, adult male mice of the outbred CD1 strain were used (n = 50 in total, Anlab, Prague, Czech Republic). Mice were housed in groups of five in standard cages with controlled 12/12 hours light/dark cycle, constant temperature (22±2°C) and humidity (45–65%). All animals had free access to water and standard rodent chow during the whole experiment.

### Design of experiment

After 2 weeks of acclimation, mice were randomly divided into 4 groups. To model AKI, mice were bilaterally nephrectomized in one surgical session (BNX group, n = 12), as described by Hoke et al., 2007 (S1 Fig) [22]. Briefly, animals were anesthetized using ketamine and xylazine (100 mg/kg and 10 mg/kg, respectively). A midline incision was made and the kidneys were exposed and decapsulated. Renal pedicles were tied off with a suture and then removed. The incision was closed with an absorbable suture. Animals in the control group (CTRL, n = 14) underwent sham surgery. After 24 hours, blood was collected from the retro-orbital plexus into K_3_EDTA tubes. Blood samples were centrifuged at 5000 g for 5 minutes. Salivation was induced using intraperitoneal application of pilocarpine (Unimed Pharma, Bratislava, Slovakia, 0.5 mg/kg). Animals were sacrificed under general anesthesia. Plasma and saliva samples were stored at -20°C until further analyses.

To induce CKD in the adenine group (n = 13), 100 mg/kg of adenine hydrochloride hydrate (Sigma Aldrich, Münich, Germany) was applied by daily intraperitoneal injection for 3 weeks (S1 Fig). Mice in the control group were treated in the same manner but received saline (CTRL group, n = 12). After 3 weeks, animals were sacrificed under general anesthesia. Blood and saliva were collected, processed and stored as mentioned above in AKI animals. Plasma and saliva samples were stored at -20°C until further analysis.

### Biochemical analysis

Creatinine and urea concentrations in plasma and saliva samples were measured using commercially available spectrophotometric assays (Creatinine Serum Low Sample Volume; Urea Nitrogen Colorimetric Detection Kit, Arbor Assays, Ann Arbor, USA). Standard protocols recommended by the manufacturer were used. For creatinine measurement, 15 microliters of samples and standards were mixed with 15 microliters of Assay Diluent in 384 wells plate. Afterwards, 60 microliters of DetectX Creatinine Reagent were added. After incubation for 1 minute and 30 minutes at room temperature, absorbance was measured at 490 nm. Sensitivity of this kit was 0.081 mg/dl. Intra-assay variability was 10% and inter-assay variability was 25%, respectively. Mean recovery was 106%. For urea measurement, saliva samples were diluted 1:1 and plasma samples were diluted twenty times with distilled water. Fifty microliters of diluted samples and standards were mixed with Color Reagent A and Color Reagent B. Absorbance was measured at 450 nm after incubation for 30 minutes. Sensitivity for urea was determined as 0.030 mg/dl. Intra-assay variability was 1.5% and inter-assay variability was 5%, respectively. Mean recovery was 98%.

To reduce variability in the analyses, all samples in each experiment were measured in a single assay.

### Statistical analysis

GraphPad Prism 6.01 (GraphPad Software Inc, La Jolla, USA) was used for statistical analysis of the obtained data. Student t-test was used for the evaluation of the differences between experimental and control groups. Correlations between plasma and salivary levels of creatinine and urea were evaluated using Pearson's correlation analysis. All values are expressed as mean + SD. P values below 0.05 were considered statistically significant.

## Results

**Fig 1:**
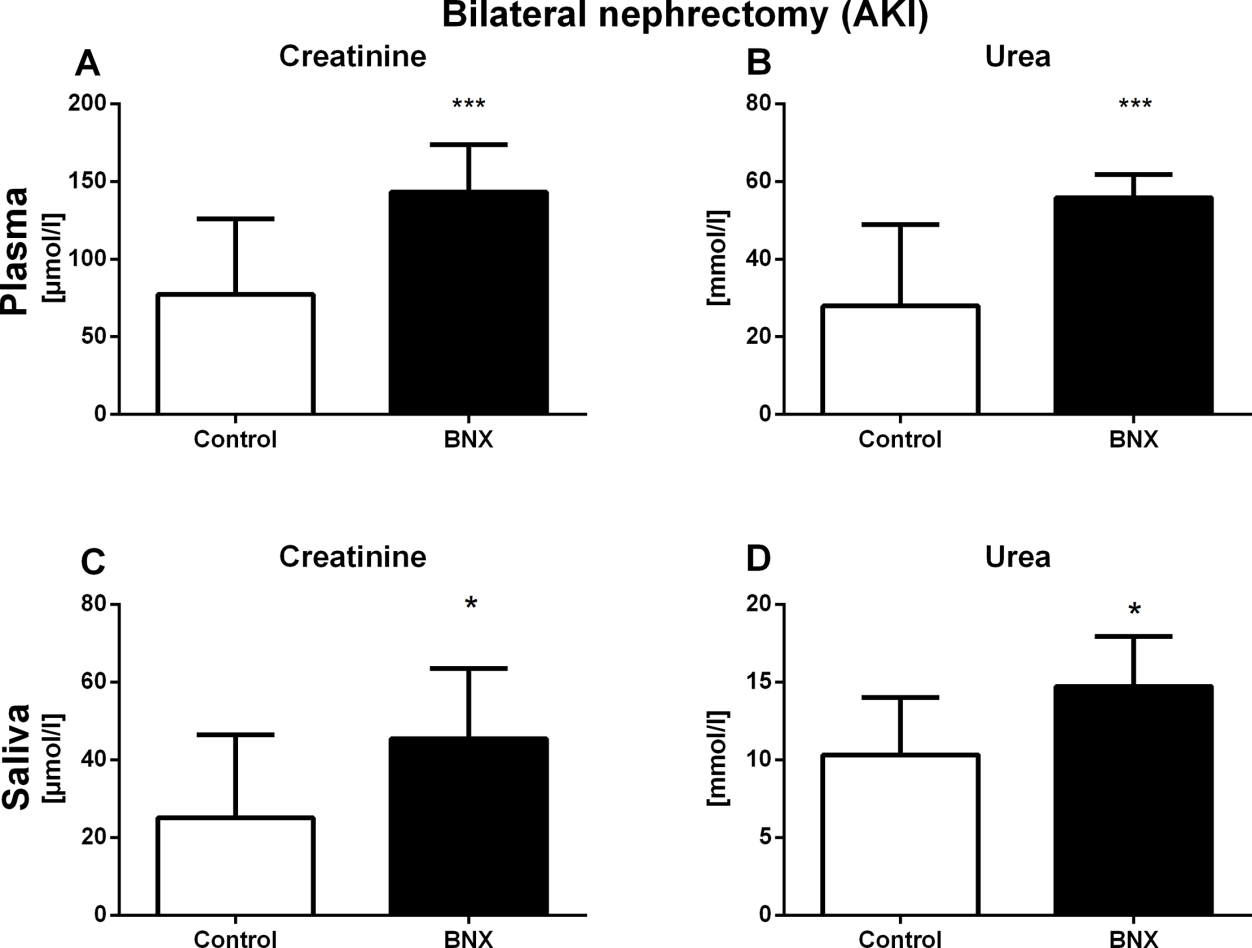
Plasma and salivary concentrations of creatinine and urea in AKI. Concentration of plasma (A) creatinine and (B) urea, and salivary (C) creatinine and (D) urea 24 hours after AKI induction. Results are expressed as mean + SD. * denotes p<0.05 and *** denotes p<0.001 in comparison to control group (by Student’s unpaired t test, n = 10 for each group).

Plasma creatinine concentrations were by 85% higher in BNX group compared to the control group (t = 4.00, p<0.001; S1 Table). Similarly, plasma concentrations of urea were significantly higher in BNX group when compared to the control group (by 99%, t = 4.42, p<0.001; Fig 1A and 1B, respectively). Salivary creatinine and urea concentrations were significantly higher in BNX group in comparison to control group (by 81%, t = 2.37, p<0.05 for creatinine; by 43%, t = 2.85, p<0.05; Fig 1C and 1D respectively).

**Fig 2:**
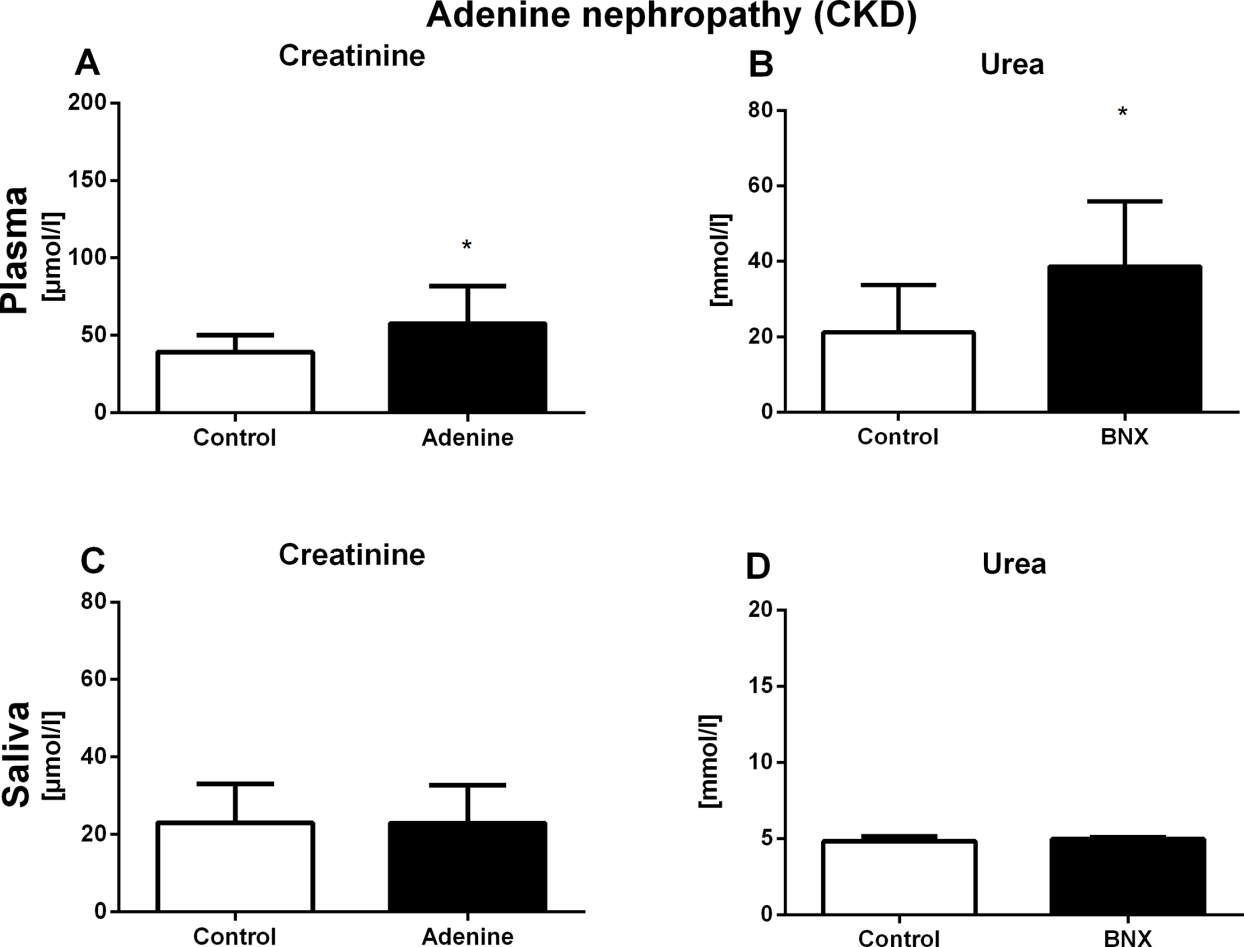
Plasma and salivary concentrations of creatinine and urea in CKD. Concentration of plasma (A) creatinine and (B) urea, and salivary (C) creatinine and (D) urea after 3 weeks of CKD induction. Results are expressed as mean + SD. * denotes p<0.05 in comparison to control group (by Student’s unpaired t test, n = 15 for each group).

Plasma creatinine concentrations were higher by 47% in the adenine group when compared to the control group (t = 2.32, p<0.05). Plasma concentrations of urea were significantly higher in adenine mice in comparison to control mice (by 82%, t = 2.58, p<0.05; Fig 2A and 2B, respectively). In the adenine nephropathy experiment, no differences were found in salivary concentrations of markers of renal function between the groups (t = 0.02, p = 0.98 for creatinine; t = 1.57, p = 0.13 for urea; Fig 2C and 2D).

**Fig 3:**
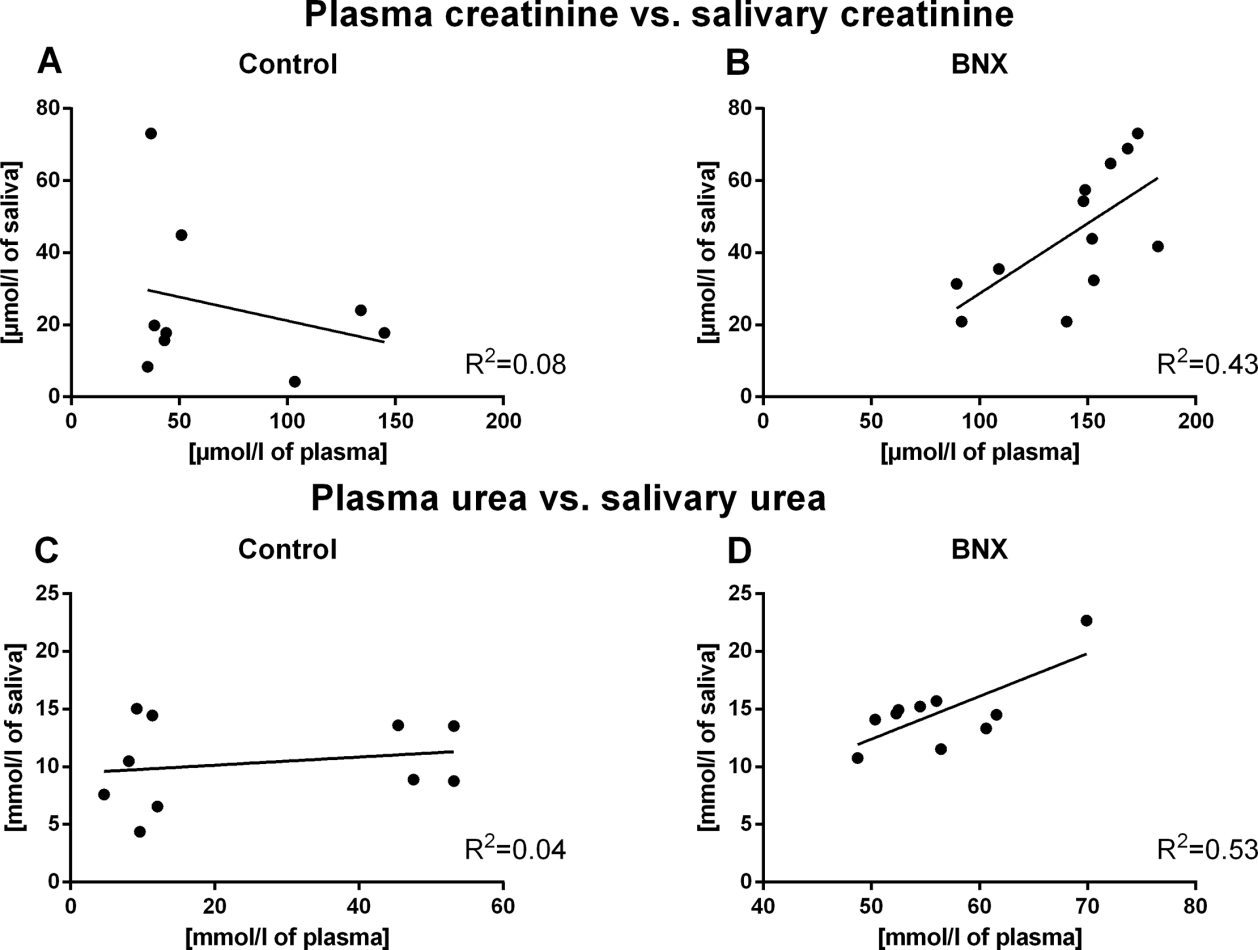
Correlation between plasma creatinine and urea and their salivary levels. Correlation between plasma creatinine and salivary creatinine levels in (A) control group (p>0.05) and (B) BNX group (p<0.05). Correlation between plasma urea and salivary urea levels in (C) control group (p>0.05) and (D) BNX group (p<0.05) (by Pearson’s correlation analysis).

**Fig 4:**
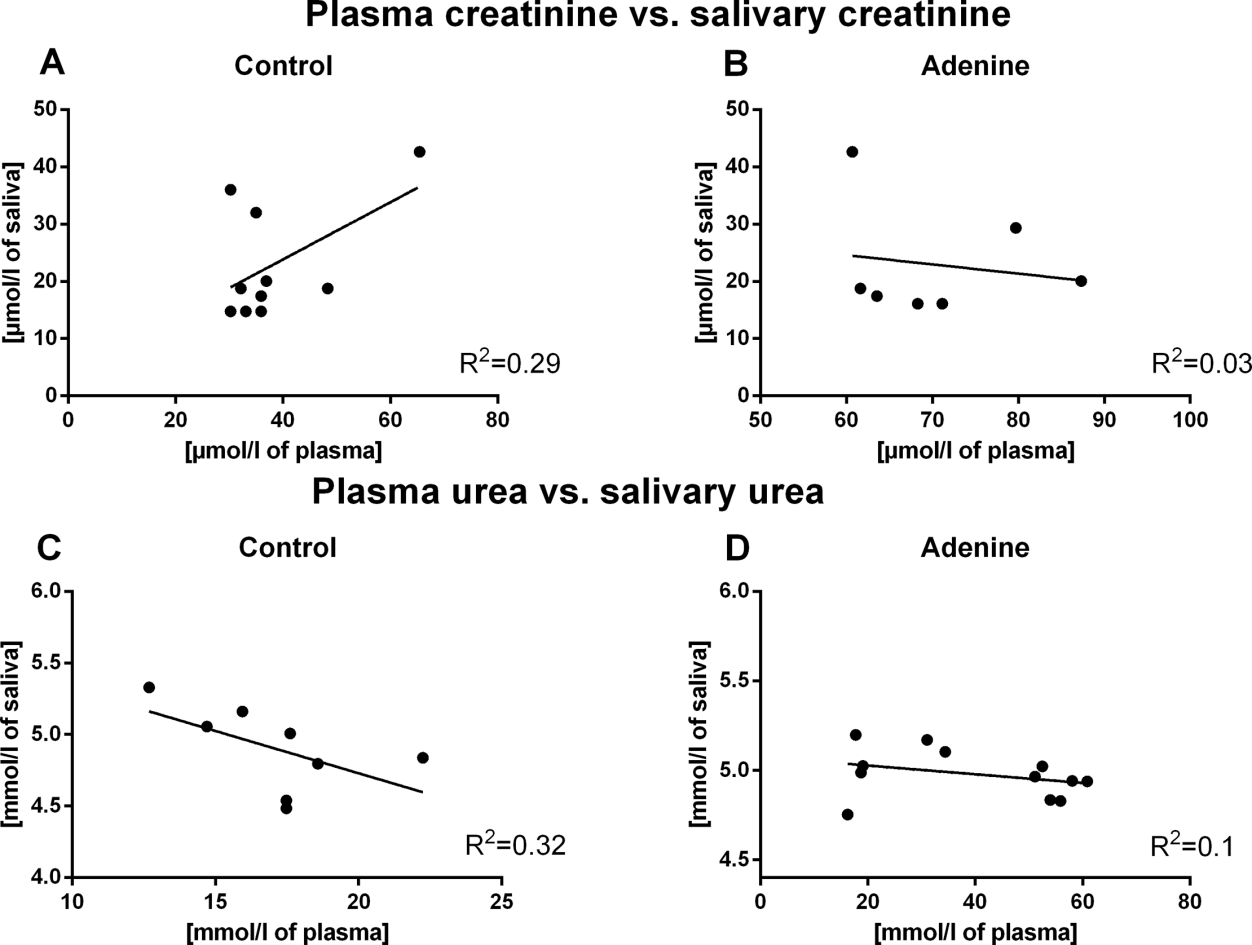
Correlation between plasma creatinine and urea and their salivary levels. Correlation between plasma creatinine and salivary creatinine levels in (A) control group (p>0.05) and (B) Adenine group (p>0.05). Correlation between plasma urea and salivary urea levels in (C) control group (p>0.05) and (D) Adenine group (p>0.05) (by Pearson’s correlation analysis).

Correlations between plasma and salivary levels of creatinine and urea in control groups were found neither in the AKI, nor in the CKD model (R = -0.28, R^2^ = 0.08, p = 0.47 for creatinine, R = 0.20, R^2^ = 0.04, p = 0.57 for urea, for the control group in the AKI experiment, Fig 3A and 3C; R = 0.54, R^2^ = 0.29, p = 0.11 for creatinine; R = -0.57, R^2^ = 0.32, p = 0.14 for urea; for the control group in the CKD model, Fig 4A and 4C).

Significant and positive correlations were found between plasma creatinine/urea and their salivary concentrations in the BNX group (R = 0.66, R^2^ = 0.43, p<0.05 for creatinine; R = 0.73, R^2^ = 0.53, p<0.05 for urea; Fig 3B and 3D). In adenine mice, no significant correlations between plasma and salivary levels of creatinine and urea were observed (R = -0.16, R^2^ = 0.03, p = 0.72 for creatinine; R = -0.32, R^2^ = 0.1, p = 0.30 for urea; Fig 4B and 4D).

## Discussion

In our study, plasma as well as salivary concentrations of creatinine and urea in models of AKI and CKD were determined. To the best of our knowledge, this is the first study to analyze salivary markers of renal function in mouse models of BNX and adenine nephropathy. Both models have been established successfully, as shown by significantly higher plasma creatinine and urea concentrations in comparison to control groups. Higher salivary concentrations of these makers have been observed only in saliva from bilaterally nephrectomized mice, but not in mice with induced adenine nephropathy. Accordingly, positive correlations between plasma and salivary markers of renal functions have been observed only in mice with AKI and not in controls or in mice with CKD. Thus, salivary concentrations of creatinine and urea reflect plasma concentrations only in mice with substantially decreased renal functions. The results are consistent with previous studies showing that salivary creatinine and urea reflect their plasma levels only in patients with kidney diseases, but not in healthy controls [25,26]. Studies focusing on patients with CKD have shown that the analysis of saliva might be useful to diagnose or monitor CKD and to distinguish moderate stage from terminal stage renal failure [11,15,27]. Similarly, Romero et al. (2016) have found a positive correlation between plasma and salivary urea concentrations in rats with induced CKD [28]. In contrast, our mouse model of CKD did not lead to higher salivary creatinine or urea. However, both experimental studies differ in the animal species used (rats vs mice), the induced renal failure model (5/6 nephrectomy vs adenine nephropathy), but also in the duration of the experiment (12 weeks vs 3 weeks). These aspects might explain the differences in the outcome.

We hypothesized that an increase of salivary creatinine and urea depends on the experimental model of renal failure and its severity. Adenine intake is associated with the formation of adenine precipitates that cause an occlusion of renal tubules. This leads to a gradual deterioration of renal function and accumulation of creatinine and urea [29]. BNX is a model of rapid loss of renal functions with a significant increase of serum creatinine already after 2 hours [17]. Adenine nephropathy in comparison to BNX results in worsening of the renal functions that is less rapid and less severe. The increase of plasma creatinine and urea was higher in BNX (85% for creatinine and 99% for urea) then in adenine nephropathy (47% for creatinine and 82% for urea respectively) in comparison to the corresponding control groups. It could be an explanation of the increase of salivary creatinine and urea only in BNX mice and not in mice with adenine nephropathy. The data are consistent with studies that have shown positive correlation of serum and salivary creatinine only in patients with high plasma creatinine and urea and not in healthy controls [25,30]. It is likely that an increase of salivary creatinine occurs only if plasma creatinine reaches a threshold concentration that creates a diffusion gradient [31]. This would mean that only severely diminished renal functions are reflected by an increase of salivary creatinine and urea. Salivary markers of renal functions could, thus, be used in clinical practice only for monitoring of advanced stages of renal diseases, but not for screening of their early stages.

It is known that colorimetric methods are non-specific [32]. On the other hand, the main goal of saliva usage in clinical practice is home monitoring, using dipsticks. Patients should monitor their kidney functions by observing the visible color changes of the dipstick moistened with saliva, that indicate range of concentration. Thus, patients could determine, whether their health condition deteriorated or not. It has been shown, that the monitoring of salivary markers using dipsticks could distinguish controls and patients with kidney disease [33,34]. Indeed, we think that plasma creatinine and urea need to reach some threshold to diffuse to saliva. This might be the reason of poor correlation between plasma and saliva at the lower levels of salivary urea. This assumption is strengthened by previously published paper by Raimann et al. (2016) and Evans et al. (2017). They showed better diagnostic performance of salivary urea at higher plasma urea when compared to lower levels of plasma urea concentrations [33,34]. We have not tested any urea strips, but it is very likely that this might be the reason for their worse performance at lower plasma urea levels.

To collect saliva from experimental animals, it is needed to stimulate salivation using pilocarpine. Stimulation of salivation, however, changes the composition of saliva and could, thus, influence the concentrations of various salivary markers [35]. Another limitation of this study is that the salivary concentrations were not normalized, however it is currently still questionable how to normalize concentrations of salivary markers. As the absolute concentrations of salivary creatinine and urea did not differ between CKD mice and controls, it is unlikely that any normalization would change the main outcome. It should be noted that CKD is according to Kidney disease improving global outcomes (KDIGO) defined as abnormal kidney structure or function for at least 3 months. A significant increase of plasma creatinine and urea could be observed from the stage 3 of CKD onwards. In our study we applied adenine hydrochloride hydrate only for 3 weeks as a model of CKD [36]. Since adenine nephropathy is not an ideal model of CKD, to confirm our results should be confirmed on more accurate models of CKD such as 5/6 nephrectomy [37]. The detailed dynamics of salivary creatinine and urea during the induction of renal failure is currently unknown. Therefore, further experimental studies focused on monitoring of salivary markers in different time points of development of renal disease are needed.

## Conclusion

In this study, both models–BNX and adenine nephropathy—were induced successfully as indicated by higher plasma creatinine and urea. Salivary concentrations of creatinine and urea were affected only in BNX, likely due to the rapid onset and severity of renal failure. What is the threshold for plasma creatinine to enter saliva as well as why only rapid and severe renal failure is reflected in saliva should be revealed in further studies.

## Supporting information

S1 Fig Design of the study. To induce AKI, mice underwent bilateral nephrectomy in one surgical session. Blood and saliva were collected 24 hours after surgery. To induce CKD, adenine nephropathy was induced. Blood and saliva were collected after 3 weeks administration of adenine hydrochloride hydrate. Creatinine and urea in plasma and saliva were measured using commercial kits.
(TIF)

S1 Table Minimal anonymized data set. The table contains concentrations of plasma and salivary creatinine and urea in individual mice. The data that are not showed were under the detection limit of kit.
(DOCX)
