# Supplementary figures and images for: Salivary creatinine and urea are higher in an experimental model of acute but not chronic renal disease
Source: PLoS One. 2018 Jul 6;13(7):e0200391. doi: 10.1371/journal.pone.0200391 (PMC6034877; doi:10.1371/journal.pone.0200391)

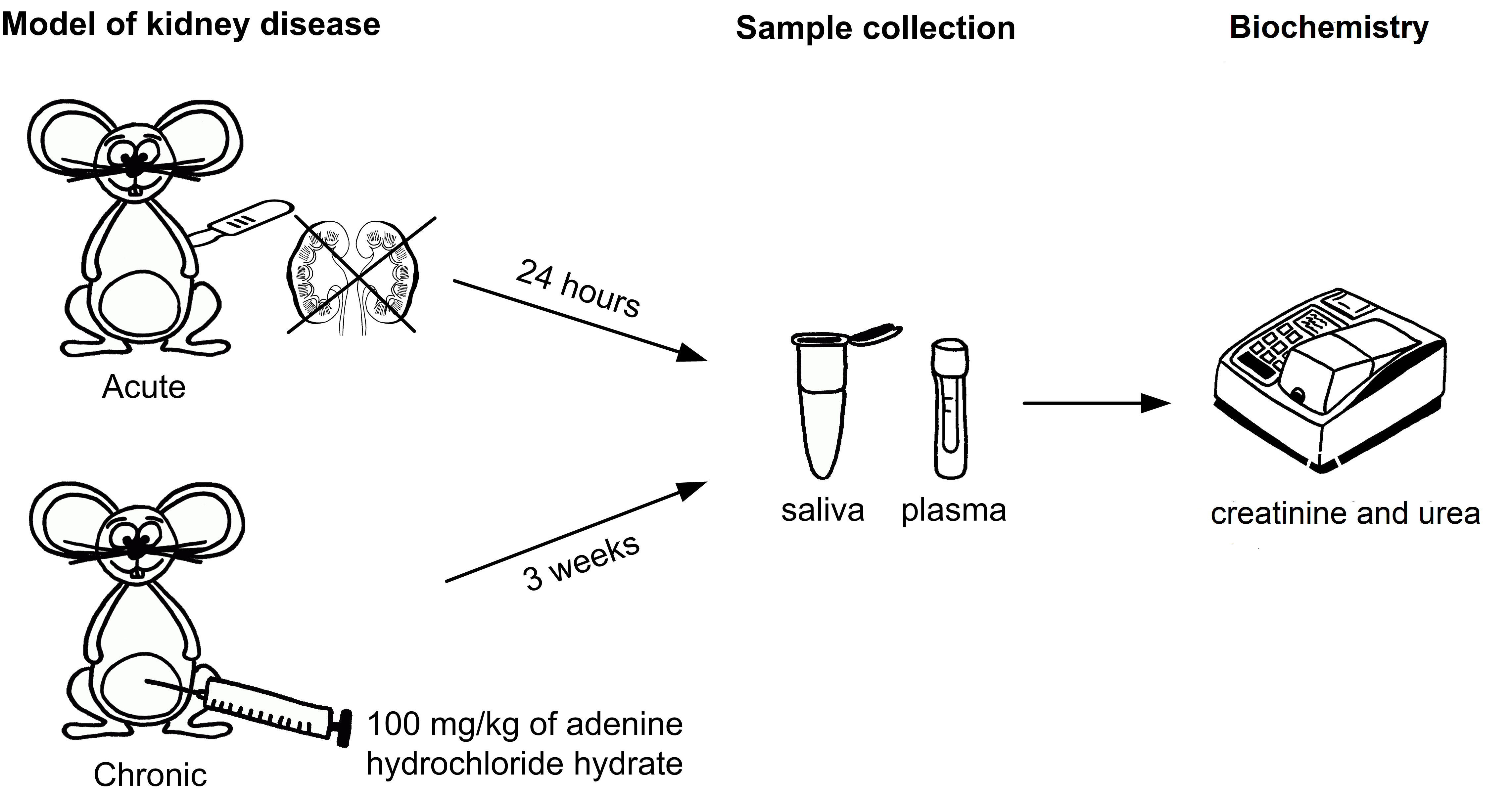

Supplement: S1 Fig — To induce AKI, mice underwent bilateral nephrectomy in one surgical session. Blood and saliva were collected 24 hours after surgery. To induce CKD, adenine nephropathy was induced. Blood and saliva were collected after 3 weeks administration of adenine hydrochloride hydrate. Creatinine and urea in plasma and saliva were measured using commercial kits. (TIF) [file pone.0200391.s001.tif]
