# Supplementary material for: Salivary creatinine and urea are higher in an experimental model of acute but not chronic renal disease
Source: PLoS One. 2018 Jul 6;13(7):e0200391. doi: 10.1371/journal.pone.0200391 (PMC6034877; doi:10.1371/journal.pone.0200391)
Supplement: S1 Table — The table contains concentrations of plasma and salivary creatinine and urea in individual mice. The data that are not showed were under the detection limit of kit. (DOCX) [file pone.0200391.s002.docx]

| Gender | Group | Plasma creatinine (µmol/l) | Salivary creatinine (µmol/l) | Plasma urea (mmol/l) | Salivary urea (mmol/l) |
| --- | --- | --- | --- | --- | --- |
| Male | CTRL | 43.21 | 44.91 | 4.64 | 7.60 |
| Male | CTRL | 38.51 | 19.86 | 8.09 | 10.49 |
| Male | CTRL | 36.95 | 73.10 | 47.58 | 8.88 |
| Male | CTRL | 103.48 | 4.20 | 53.20 | 8.77 |
| Male | CTRL | 35.38 | 8.38 | 9.62 | 4.37 |
| Male | CTRL | 144.97 | 17.77 | 53.20 | 13.54 |
| Male | CTRL | 43.99 | 17.77 | 11.35 | 14.44 |
| Male | CTRL | 51.04 | 44.91 | 45.45 | 13.59 |
| Male | CTRL | 134.01 | 24.04 | 9.19 | 15.04 |
| Male | CTRL | 58.87 | ─ | 12.05 | 6.56 |
| Male | CTRL | 54.95 | ─ | 4.40 | ─ |
| Male | CTRL | 77.65 | ─ | 42.38 | ─ |
| Male | CTRL | 83.32 | ─ | 34.33 | ─ |
| Male | CTRL | ─ | ─ | 56.43 | ─ |
| Male | BNX | 182.54 | 41.78 | 48.72 | 10.75 |
| Male | BNX | 89.39 | 31.34 | 56.43 | 11.54 |
| Male | BNX | 140.27 | 20.90 | 69.89 | 22.67 |
| Male | BNX | 152.01 | 43.87 | 52.50 | 14.92 |
| Male | BNX | 91.74 | 20.90 | 56.02 | 15.70 |
| Male | BNX | 108.96 | 35.52 | 61.57 | 14.50 |
| Male | BNX | 160.62 | 64.75 | 54.50 | 15.21 |
| Male | BNX | 148.88 | 57.44 | 50.35 | 14.08 |
| Male | BNX | 148.10 | 54.31 | 60.61 | 13.32 |
| Male | BNX | 173.15 | 73.10 | 52.30 | 14.61 |
| Male | BNX | 152.80 | 32.39 | 57.15 | ─ |
| Male | BNX | 168.45 | 68.92 | 49.58 | ─ |
| Male | CTRL | 30.24 | 36.00 | 14.71 | 5.06 |
| Male | CTRL | 49.26 | ─ | ─ | ─ |
| Male | CTRL | 65.44 | 42.64 | ─ | ─ |
| Male | CTRL | 48.31 | 18.76 | ─ | ─ |
| Male | CTRL | 35.94 | 17.43 | 22.24 | 4.84 |
| Male | CTRL | 35.94 | 14.76 | 17.48 | 4.54 |
| Male | CTRL | 30.24 | 14.78 | 12.69 | 5.33 |
| Male | CTRL | 36.90 | 20.08 | 17.48 | 4.48 |
| Male | CTRL | 34.99 | 32.02 | 18.58 | 4.80 |
| Male | CTRL | 33.09 | 14.78 | 15.95 | 5.16 |
| Male | CTRL | 32.14 | 18.76 | 17.61 | 5.01 |
| Male | CTRL | ─ | ─ | ─ | ─ |
| Male | AD | 23.58 | ─ | 16.27 | 4.75 |
| Male | AD | 21.67 | ─ | 18.74 | 4.99 |
| Male | AD | 17.87 | ─ | 17.74 | 5.80 |
| Male | AD | 37.85 | ─ | 19.09 | 5.02 |
| Male | AD | 79.71 | 29.37 | 60.88 | 4.94 |
| Male | AD | 60.68 | 42.64 | 51.15 | 4.96 |
| Male | AD | 71.14 | 16.10 | 53.99 | 4.83 |
| Male | AD | 87.32 | 20.08 | 58.05 | 4.94 |
| Male | AD | 61.63 | 18.76 | 55.90 | 4.83 |
| Male | AD | 63.53 | 17.43 | 31.06 | 5.17 |
| Male | AD | 68.29 | 16.10 | 34.44 | 5.10 |
| Male | AD | 79.71 | ─ | 52.50 | 5.02 |
| Male | AD | 75.90 | ─ | 32.46 | ─ |
